# Differential Effect of TRPV1 Modulators on Neural and Behavioral Responses to Taste Stimuli

**DOI:** 10.3390/nu16223858

**Published:** 2024-11-12

**Authors:** Mee-Ra Rhyu, Mehmet Hakan Ozdener, Vijay Lyall

**Affiliations:** 1 Department of Food Science and Biotechnology, Sejong University, Seoul 05006, Republic of Korea; mrrhyu@sejong.ac.kr; 2 Monell Chemical Senses Center, Philadelphia, PA 19104, USA; ozdener@monell.org; 3 Department of Physiology and Biophysics, Virginia Commonwealth University, Richmond, VA 23298, USA

**Keywords:** transient receptor potential vanilloid type 1 channel, capsaicin, resiniferatoxin, kokumi taste peptides, salt taste, sour taste, sweet taste, bitter taste, umami taste

## Abstract

In our diet, we ingest a variety of compounds that are TRPV1 modulators. It is important to understand if these compounds alter neural and behavioral responses to taste stimuli representing all taste qualities. Here, we will summarize the effects of capsaicin, resiniferatoxin, cetylpyridinium chloride, ethanol, nicotine, *N*-geranyl cyclopropylcarboxamide, Kokumi taste peptides, pH, and temperature on neural and behavioral responses to taste stimuli in rodent models and on human taste perception. The above TRPV1 agonists produced characteristic biphasic effects on chorda tympani taste nerve responses to NaCl in the presence of amiloride, an epithelial Na^+^ channel blocker, at low concentrations enhancing and at high concentrations inhibiting the response. Biphasic responses were also observed with KCl, NH_4_Cl, and CaCl_2_. In the presence of multiple stimuli, the effect is additive. These responses are blocked by TRPV1 antagonists and are not observed in TRPV1 knockout mice. Some TRPV1 modulators also increase neural responses to glutamate but at concentrations much above the concentrations that enhance salt responses. These modulators also alter human salt and glutamate taste perceptions at different concentration ranges. Glutamate responses are TRPV1-independent. Sweet and bitter responses are TRPV1-independent but the off-taste of sweeteners is TRPV1-dependent. Aversive responses to acids and ethanol are absent in animals in which both the taste system and the TRPV1-trigeminal system are eliminated. Thus, TRPV1 modulators differentially alter responses to taste stimuli.

## 1. Introduction

Among members of the transient receptor potential cation channel family, the least amount of information is available on the role of transient receptor potential vanilloid type 1 channel (TRPV1) in taste [1,2]. This is partly due to the fact that TRPV1 expression in taste receptor cells (TRCs) is species-dependent. TRPV1 is not expressed in rodent TRCs but it is expressed in human taste cells [3,4]. In addition, TRPV1 modulators produce varying effects on different taste nerves to taste stimuli depending upon the taste receptive field being stimulated [5]. TRPV1 is highly expressed in trigeminal nerves surrounding the taste buds [2]. TRPV1 expressed in a subset of TRCs can potentially directly interact with a specific taste quality. Alternately, TRPV1 expressed in the trigeminal nerves can contribute to the aversive responses to some taste stimuli [6]. Activation of TRPV1 in trigeminal nerves can indirectly alter specific taste responses by releasing peptides (substance P and calcitonin gene-related peptide) [7]. The aim of this review is to first describe the taste transduction mechanism of each taste quality and then summarize the effect of TRPV1 modulators on neural and behavioral responses to taste stimuli representing all taste qualities. In our diet, we ingest a variety of compounds that are potential TRPV1 modulators. Therefore, it is important to understand how these compounds affect taste responses to salty, sweet, umami, bitter, and sour stimuli. Some TRPV1 modulators can be useful in devising strategies to decrease excessive consumption of high salt, ethanol, and nicotine.

### TRPV1

TRPV1 is a polymodal non-selective cationic channel. It is a tetrameric protein made up of four monomers, with each monomer harboring six transmembrane segments (S1–S6) with cytoplasmic *C*- and *N*-terminal domains and a pore region between the S5 and S6 domains [8]. A large number of ligands activate the channel exogenously and endogenously. These include capsaicin (Cap), resiniferatoxin (RTX), piperine, gingerol, zingerone, camphor, eugenol, ethanol, some toxins in venom, noxious temperatures (>43 °C), acid pH, divalent cations (Mg^2+^ and Ba^2+^), *N*-arachidonoylethanolamine (anandamide), 2-arachidonoylglycerol, *N*-arachidonoyl dopamine, *N*-oleoyldopamine, ATP, lipoxygenase products, and monoacylglycerols. When multiple stimuli are presented together, TRPV1 activation is potentiated. TRPV1 is expressed in neurons and glial cells, and in non-neuronal tissues, such as heart, liver, lung, kidney, adipose tissue, skeletal muscle, and intestine. Upon activation, the channel allows Na^+^ and Ca^2+^ influxes that depolarize cells [9]. TRPV1 is regulated by intracellular adenosine-5′-triphosphate (ATP), phosphoinositides [10], protein kinas A (PKA) [11], and Ca^2+^ [12]. In cells expressing TRPV1 and transient receptor potential ankyrin 1 (TRPA1) ion channel, TRPA1 modulators may also augment TRPV1 activity [13].

## 2. Effect of TRPV1 Modulators on Neural and Behavioral Response to Sour Taste Stimuli

Sour taste is elicited by acidic stimuli and produces an aversive response. Recordings of the rat chorda tympani (CT) taste nerve, while the anterior fungiform receptive field was under lingual voltage-clamp, revealed that HCl responses are voltage-sensitive [14]. Relative to zero current clamp, the magnitude of the HCl CT response increased at −90 mV and decreased at +90 mV. Zn^2+^ inhibited the HCl CT response in a concentration-dependent manner, and eliminated the voltage sensitivity (±90 mV) of the response. Zn^2+^ only partially inhibited the CT response to H_3_PO_4_ but did not inhibit response to acetic acid. HCl and H_3_PO_4_ generate H^+^ externally while acetic acid is membrane-permeable and decreases TRC pH_i_. Increasing TRC cAMP in vivo increased the magnitude of the HCl CT response that demonstrated voltage dependence over all voltages between +90 mV and −90 mV [14]. These results suggested that, in the fungiform taste receptive field, a Zn^2+^-sensitive proton channel that is activated by cAMP is involved in the detection of H^+^ in TRCs.

Polycystin-2-like 1 (PKD2L1) channel is expressed exclusively in type III TRCs in the mouse. Animals in which PKD2L1-expressing cells in the taste buds were eliminated did not elicit neural responses to sour taste stimuli [15,16]. However, mice lacking PKD2L1 and/or polycystin-1-like-3 or transient receptor potential channel (PKD1L3), still demonstrated robust taste responses to acids [17,18]. This indicated that PKD2L1/PKD1L3 channels are markers of type III TRCs but are not involved in detecting sour taste stimuli. The search for the elusive proton channel was upended by two research groups by demonstrating the expression and function of Otopetrin-1 (OTOP1), a proton-selective channel, in PKD2L1-positive TRCs [19]. OTOP1 is required for a decrease in pH_i_ in Type III TRCs and is inhibited by Zn^2+^. This indicates that a decrease in TRC pH_i_ is the proximate signal for sour taste transduction [20].

Sour-sensing cells also detect CO_2_. CT responses to CO_2_ are voltage sensitivity. Compared with open-circuit conditions, CO_2_ CT responses were enhanced at −60 mV and suppressed at +60 mV [20]. CO_2_-induced decrease in TRC pH_i_ and CT response were inhibited by membrane-permeable blockers of carbonic anhydrases, MK-417 and MK-927. In subsequent studies, carbonic anhydrase-4 (Car4) was identified in type III TRCs expressing PKD2L1 and OTOP1 [6,21]. Car4 KO mice had significantly diminished CT response to CO_2_. Most importantly, when tetanus toxin light chain was trans-genetically targeted to type III TRCs, it not only prevented the neurotransmitter release from these TRCs but also selectively and completely abolished responses to sour stimuli and CO_2_ [21]. Prodynorphin-expressing sour-responding neurons in the rostral nucleus of the solitary tract receive direct and selective input from Proenkephalin (Penk)-expressing sour ganglion neurons that, in turn, receive input from type III cells [6]. Thus, a subset of TRCs and ganglion cells are dedicated to sour taste sensing.

Stimulating with HCl containing RTX, a potent capsaicin-like TRPV1 agonist, did not alter rat CT responses to HCl [22]. I-RTX (a high affinity TRPV1 antagonist) also did not affect mouse CT responses to acetic acid, citric acid, and HCl but significantly suppressed mouse GL responses to the above acids. Mouse SL nerve responded in a concentration-dependent manner to the above acids. The responses to acetic acid, but not to the other acids were inhibited by I-RTX. These results suggested that TRPV1 is likely involved in responses to acids in the posterior oral cavity and larynx. This high degree of responsiveness to acetic acid may be responsible for the oral burning sensation of vinegar [5].

Genetic ablation of PKD2L1-expressing TRCs or generating OTOP1 KO mice did not alter the aversive behavior of mice to acids. In OTOP1 KO mice in which trigeminal TRPV1 neurons were ablated, mice exhibited a major loss of behavioral aversion to acid [6]. Thus, the trigeminal system and the taste system work in concert to evoke aversive responses to acids. However, CO_2_ responses are entirely dependent upon type III cells [21]. *CO_2_ specifically activates a subpopulation of trigeminal neurons that expresses TRPA1 but does not activate TRPV1* [23].

## 3. Effect of TRPV1 Modulators on Neural and Behavioral Responses to NaCl, KCl, NH_4_Cl, and CaCl_2_

### 3.1. TRPV1 and Taste Responses to NaCl

Salt taste is transduced by an amiloride (Am)-sensitive pathway that is Na^+^-specific and an Am-insensitive pathway that is cation non-selective. TRPV1 modulators differentially alter both salt-sensing pathways in humans and rodent models.

### 3.2. TRPV1 and ENaC-Dependent Na^+^-Specific Salt Taste

Am-sensitive Na^+^-specific salt taste is detected by a subset of Type II fungiform TRCs that express αENaC (epithelial Na^+^ channel), phospholipase C β2 (PLCβ2), IP3 (inositol triphosphate), CALHM3 (Ca^2+^ homeostasis modulator 3), and a transcription factor skin head, SKN-1a. These cells do not express TRPM5 (Transient Receptor Potential Cation Channel Subfamily M Member 5) and GNAT3 (guanine nucleotide-binding protein G(t) subunit alpha-3). Na^+^ entry through ENaC induces a suprathreshold depolarization for action potentials driving voltage-dependent, Ca^2+^-independent neurotransmitter release via the CALHM1/3 channel synapse [24,25,26]. ENaC is composed of α, β, and γ subunits but these subunits have segregated expression in mouse taste buds. Thus, the exact subunit composition of the functional ENaC channels in rodent TRCs is unclear [27]. In geniculate ganglia, Early growth response protein 2 (Egr2)-expressing neurons receive input from cells that detect appetitive NaCl concentrations [6]. Thus, appetitive salt responses are detected by dedicated TRCs and geniculate ganglia neurons [28].

**Figure 1:**
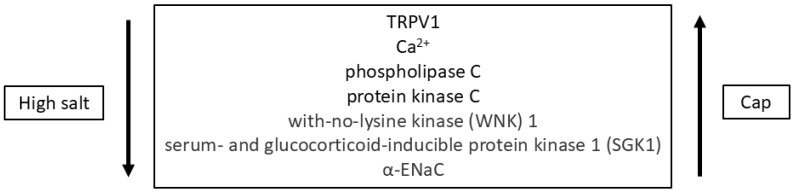
A proposed mechanism for TRPV1-dependent regulation of ENaC expression in HBO cells. High salt inhibits TRPV1 and Cap activates TRPV1, resulting in changes in [Ca^2+^]_i_. Downstream from [Ca^2+^]_i_, several intracellular effectors are most likely inhibited or activated that alter ENaC expression [32].

In contrast to rodent TRCs [2], TRPV1 mRNA was detected in cultured human taste cell lysates [3]. In human fungiform taste cells (HBO cells) [4], TRPV1 mRNA expression and TRPV1 antibody was localized in HBO cells expressing δ-ENaC subunits and δ-ENaC subunits co-localized with gustducin and PLCβ_2_. Both α-ENaC and γ-ENaC subunits also co-localized with PLCβ_2_. The δ-ENaC subunit expressing HBO cells also expressed components of the renin–angiotensin–aldosterone system (RAAS) and G-protein-coupled estrogen receptor, signaling molecules that regulate ENaC expression and taste responses to NaCl [29,30,31]. Some ENaC regulators are most likely present in a complex and changes in the expression of one or more regulators can alter the expression of other effectors. Culturing HBO cells in media containing high salt induced an increase in δ-ENaC mRNA and protein and decreased TRPV1 mRNA expression. On the other hand, culturing them in media containing 2.5 μM Cap increased the expression of TRPV1 [4]. In kidney cortical collecting duct cells, both high salt and Cap modulate TRPV1-dependent ENaC expression [32]. As summarized in Figure 1, high salt inhibits and Cap activates TRPV1 and alters [Ca^2+^]_i_. Downstream from [Ca^2+^]_i_, several intracellular signaling components are either inhibited or activated that modulate ENaC expression [32]. Polymorphisms of the *TRPV1* gene are associated with alterations in salty taste sensitivity and salt preference [33]. Human salt-sensing TRCs express TRPV1 and δ-ENaC subunit. The effect of Cap in mitigating high salt-induced changes in ENaC expression in human taste cells may be relevant in reducing salt intake in humans [34,35]. Acutely stimulating with NaCl solutions containing Cap or RTX does not produce effects on the Am-sensitive NaCl CT response [22]. In HBO cells, Arginyl dipeptides increase the frequency of NaCl-elicited responses via ENaC α and δ subunits in HBO cells [36] and enhance saltiness of 50 mM NaCl in human sensory evaluation [37].

In some human subjects, lingual surface potential induced by oral NaCl was sensitive to Am and quantitatively correlated with the perceived salt taste intensity [38]. These studies support the role of ENaC in human salt taste. However, it is not clear if ENaC is the predominant salt taste receptor in humans and its contribution to overall human salt taste is difficult to evaluate [39]. In human lingual epithelium, δ subunit expression is much smaller compared to the expression of other subunits (α = β > γ » δ) [40], raising a question regarding the variability of the Am-sensitive and Am-insensitivity components of the human salt taste responses.

## 4. TRPV1 and Am-Insensitive and Cation Non-Selective Pathway That Detects NaCl, KCl, NH_4_Cl, and CaCl_2_

A variety of compounds with very different structures modulate Am-insensitive salt responses in rodent models (Table 1).

### 4.1. Cap, RTX, pH, and Temperature

TRPV1 modulators alter neural responses to NaCl, KCl, NH_4_Cl, and CaCl_2_ [5]. I-RTX (1–100 nM), a potent TRPV1 antagonist, decreased mouse CT responses to NaCl, KCl, and NH_4_Cl. In the GL nerve, I-RTX significantly suppressed responses to all salts. In the SL nerve, I-RTX did not inhibit responses to the above salts. In the CT nerve, I-RTX can alter NaCl responses via Am-sensitive and/or Am-insensitive pathways, while in the GL nerve the effects of I-RTX most likely are restricted to its effect on the Am-insensitive pathway(s). The observation that I-RTX suppressed responses to NaCl in both CT and GL nerves suggests that TRPV1 is directly or indirectly involved in regulating salt responses in rodents [5].

#### 4.1.1. Biphasic Effects on NaCl CT Responses

Cap (or RTX) when mixed with the NaCl stimulus did not alter the magnitude of the Am-sensitive, ENaC-dependent component of rat NaCl CT response [22]. In the presence of Am or benzamil (Bz; a more potent blocker of ENaC), adding Cap (5–200 μM) or RTX (0.1–10.0 μM) produced a biphasic response in rat NaCl CT response. CT response was enhanced at low concentrations, and at high concentrations the response decreased from its maximum value. Cap and RTX increased the maximum CT response by more than 200%. At the highest concentrations used, the response decreased to baseline (Table 1). Capsazepine (CZP) and *N*-(3-methoxyphenyl)-4-chlorocinnamide (SB-366791), TRPV1 antagonists, inhibited the effects of Cap and RTX. The Am-insensitive NaCl CT responses are voltage-sensitive and the voltage sensitivity increased in the presence of RTX concentrations that enhanced the CT response.

Varying temperature between 23 °C and 55.5 °C produced a biphasic effect on the Am-insensitive NaCl CT responses, with maximum enhancement obtained at 42 °C (Table 1). RTX increased the CT response at 23 °C and shifted the temperature curve to the left in a dose-dependent manner. In the absence of RTX, the Am-insensitive NaCl CT responses were not sensitive to changes in the pH of the stimulating solutions (pH 2–10) but in the presence of RTX demonstrated a bell-shaped curve as a function of pH (Table 1) [22]. The Am-insensitive NaCl CT responses were regulated by phosphatidylinositol 4,5-bisphosphate (PIP2) [41], intracellular Ca^2+^, protein kinase C, and calcineurin [42]. An increase in intracellular PIP_2_ inhibited the control CT response and decreased its sensitivity to RTX. On the other hand, a decrease in intracellular PIP_2_ enhanced the control Am-insensitive NaCl CT response, increased its sensitivity to RTX stimulation, and inhibited the desensitization of the CT response at high RTX concentrations [41].

In TRPV1 KO mice, Bz inhibited the NaCl CT response to baseline [43]. TRPV1 KO mice did not elicit an increase in the Am-insensitive CT responses above the rinse baseline value at all RTX concentrations tested [41]. It was previously *hypothesized* that the Am-insensitive channel is a variant of TRPV1 (TRPV1t). However, TRPV1 is not expressed in rodent TRCs. Thus, at present, the identity of this channel in the fungiform-receptive field remains unknown.

In behavioral assay, wildtype and TRPV1 KO mice in the absence and presence of Am did not show any differences in their responses to NaCl [43]. In hindsight, this is not so surprising, as inhibition of CT nerve responses to some taste stimuli do not always correlate with the behavioral responses in animals [6,19,44]. Am-insensitive NaCl responses have been suggested to reside in subsets of bitter, sour, and sweet cells [45,46,47,48] and, thus, may involve several different receptors and pathways.

#### 4.1.2. Biphasic Effects on KCl, NH_4_Cl, and CaCl_2_ CT Responses

Cap and RTX also produced similar biphasic effects on CT response to KCl, NH_4_Cl, and CaCl_2_, suggesting that this conductive pathway is a non-specific cation channel that allows Na^+^, K^+^, NH_4_^+^, and Ca^2+^ ion flux across the channel [22].

At present, it is not clear how TRPV1 agonists and antagonists modulate the Am-insensitive responses in rodent TRCs in the absence of TRPV1 expression. One possibility that needs to be further explored is that the activation of TRPV1 in trigeminal nerves may indirectly alter Am-insensitive NaCl taste receptors in sweet, bitter, or salty cells by releasing substance P and calcitonin gene-related peptide [7]. Calcitonin gene-related peptide can then act on the calcitonin gene-related peptide receptor expressed in Type III cells, that, presumably, also harbor the Am-insensitive salt taste receptor(s) [45,46].

Human salt taste is largely Am-insensitive [39,49,50]. In part, this is due to the expression of an additional ENaC subunit, the δ subunit, and the observation that, unlike the αβγ ENaC channel, the δβγ ENaC channel is Am-insensitive [51].

More recently, OTOP1 has been shown to be involved in NH_4_Cl sensing [52]. It is suggested that NH_4_Cl taste may be a distinct taste from the five primary taste qualities. Zn^2+^ inhibited CT nerve responses to NH_4_Cl in a dose-dependent manner. Gustatory nerve responses to NH_4_Cl were strongly attenuated or eliminated in OTOP1 KO mice. In polarized rat taste bud cells, apical NH_4_Cl produces intracellular alkalinization. In HEK-293 cells co-transfected with *mOTOP1* and pHlourin, a pH-sensitive variant of green fluorescent protein, NH_4_Cl induced an increase in pH_i_ paralleling its ability to evoke OTOP1 currents. Aversion of mice to NH_4_Cl was diminished in Skn-1a KO mice lacking Type II TRCs, but was entirely abolished in a double KO mouse model with OTOP1. Although TRPV1 is activated by intracellular alkalinization following exposure to NH_4_Cl/NH_3_ [53], the aversive NH_4_Cl response is not dependent upon the trigeminal system and resides entirely in Type II and Type III cells.

### 4.2. N-Geranyl Cyclopropyl-Carboxamide (NGCC)

NGCC enhanced Ca^2+^ influx in hTRPV1-expressing cells in a dose-dependent manner that was significantly attenuated by ruthenium red (RR), a non-specific blocker of TRP channels, and CZP, a specific antagonist of TRPV1, implying that NGCC directly activates hTRPV1 [54]. NGCC enhanced rat CT response to NaCl+Bz between 1 and 2.5 μM and inhibited it above 5 μM (Table 1). In the presence of a TRPV1 blocker, SB-366791, both NaCl+Bz and NaCl+Bz+NGCC CT responses were inhibited to baseline. No NaCl+Bz CT response was observed in TRPV1 KO mice in the absence or presence of NGCC. NGCC enhanced human salt taste intensity of fish soup stock containing 60 mM NaCl at 5 and 10 μM and decreased it at 25 μM [55]. Thus, both neural and behavioral responses to NGCC are biphasic.

### 4.3. Ethanol and Nicotine

At concentrations < 50%, ethanol enhanced CT responses to KCl and NaCl+Bz, while at ethanol concentrations > 50%, the CT responses were inhibited (Table 1). RTX and elevated temperature increased the sensitivity of the CT response to ethanol in salt-containing media, and SB-366791 inhibited the effect of ethanol, RTX, and elevated temperature on the CT responses. TRPV1 KO mice demonstrated no Bz-insensitive NaCl CT response and no sensitivity to ethanol [56].

At concentrations < 0.015 M, nicotine enhanced and at >0.015 M inhibited CT responses to KCl and NaCl+Bz (Table 1). Nicotine produced maximum enhancement in the NaCl+Bz CT response at pH_o_ between 6 and 7. RTX and elevated temperature increased the sensitivity of the CT response to nicotine in salt-containing media, and SB-366791 inhibited these effects. TRPV1 KO mice demonstrated no NaCl+Bz CT response and no sensitivity to nicotine, RTX, and elevated temperature [57]. At pH_o_ > 8, the apical membrane permeability of nicotine was increased significantly, resulting in increase in TRC pH_i_ and volume, activation of ENaC, and enhancement of the Am-sensitive NaCl CT response. At pH_o_ > 8, nicotine also inhibited the phasic component of the HCl CT response. These results suggest that the effects of nicotine on ENaC and the phasic HCl CT response arise from increase in TRC pH_i_ and volume.

### 4.4. Kokumi Peptides

Maillard-reacted peptides (MRPs) are generated during cooking a wide range of foods containing proteins/peptides and carbohydrates. During cooking, covalent bonds between carbonyl groups and free amino groups are formed. Kokumi peptides include γ-glutamate (Glu), and other peptides containing γ-Glu such as γ-Glu-Ala, γ-Glu-Val, γ-Glu-Cys, γ-Glu-aminophenyl-Gly, and γ-Glu-Val-Gly [58,59,60,61]. These peptides activate calcium-sensing receptor (CaSR) [62] expressed in a subset of taste bud cells. CaSR is activated by cations (Ca^2+^ and Gd^3+^), peptides, and polyamines and transmits its signal through Gαq/11 proteins, PLCβ, and release of [Ca^2+^]_i_ via activation of IP3 receptor channels in the endoplasmic reticulum. CaSR is inhibited by NPS-2143 [63].

MRPs and γ-glutamyl peptides also modulate salty taste [64,65]. We synthesized MRPs by conjugating a peptide fraction (1000–5000 Da) purified from soy protein hydrolysate with galacturonic acid (GalA), glucosamine, xylose (Xyl), fructose, or glucose [66]. In mixtures containing NaCl, MRPs did not alter the Am-sensitive NaCl CT responses. In a patch-clamp study on rat fungiform taste cells, kokumi-active tripeptides, glutathione, and γ-Glu-Val-Gly did not alter ENaC activity in taste cells [67].

In contrast, GalA-MRP added to NaCl+Bz stimulating solution increased the CT response between 0.1% and 0.25% and inhibited it above 0.5% (Table 1). The effectiveness of MRPs as salt taste enhancers varied with the conjugated sugar moiety: galacturonic acid = glucosamine > xylose > fructose > glucose. Elevated temperature, RTX, capsaicin, and ethanol produced additive effects on the NaCl CT responses in the presence of MRPs. SB-366791 inhibited the NaCl+Bz CT response in the absence and presence of MRPs. TRPV1 KO mice demonstrated no Am-insensitive NaCl CT response in the absence or presence of MRPs [66].

The concentrations at which MRPs enhanced human salt taste were significantly lower. In human subjects, between 0.0025% and 0.01%, both GalA-MRP and Xyl-MRP increased, and above 0.01% suppressed the salt taste intensity. In the presence of 0.01% GalA-MRP and Xyl-MRP, the median effective salt concentration was maximally enhanced by 5.9% and 3.5%, respectively. The increase in salt intensity in the presence of GalA-MRP was also enhanced at 45 °C relative to 30 °C. In mixtures containing 0.01% GalA-MRP+4% ethanol, the perceived salt intensity was greater than that of 4% ethanol alone. However, the perceived salt taste intensity was less than that of GalA-MRP alone. Thus, in the presence of MRPs, elevated temperature and ethanol alter human salt taste perception [66].

Kokumi taste-active and -inactive peptide fraction (500–10,000 Da) were isolated from mature (FII_m_) and immature (FII_im_) Ganjang, a typical Korean soy sauce. Only FII_m_ (0.1–1.0%) produced a biphasic effect in rat CT responses to NaCl+Bz (Table 1). Both elevated temperature (42 °C) and FII_m_ produced synergistic effects on the NaCl+Bz CT response. At 0.5%, FII_m_ produced the maximum increase in NaCl+Bz CT response and enhanced salt taste intensity in human subjects [64].

In mice, adding increasing concentrations of FII_m_ (0.1 to 1%) to 100 mM NaCl solutions in the absence and presence of 10 µM Am produced biphasic changes in NaCl preference, increasing it at 0.25% and lowering it at higher concentrations. FII_m_ maximally enhanced NaCl preference at 0.25% relative to NaCl alone and above 0.25% FII_m_ was significantly less than its maximum value. In the presence of 10 µM Am, the maximum increase in NaCl preference was observed at 0.5% FII_m_ and above 0.5% FII_m_ was significantly less than its maximum value. There was no change in NaCl preference when equivalent concentrations of the FII_im_ were added to the test solutions containing 100 mM NaCl or 100 mM NaCl+10 µM Am. These behavioral responses to NaCl correlate with the biphasic effects of FII_m_ concentrations on Am-insensitive NaCl CT responses [64].

Similar to hTRPV1, NGCC enhanced Ca^2+^ influx in hTRPA1-expressing cells (EC_50_ = 83.65 µM). NGCC-induced Ca^2+^ influx in hTRPA1-expressing cells was blocked by ruthenium red and 2-(1,3-dimethyl-2,6-dioxopurin-7-yl)-N-(4-propan-2-ylphenyl)acetamide (HC-030031), a specific antagonist of TRPA1 [54]. These studies suggest that some of effects of the above compounds on NaCl may involve additional ion channels.

### 4.5. Novel Salty and Salt-Enhancing Peptides

As reviewed recently [68], some peptides derived from various food proteins can elicit salt taste themselves in the absence of Na^+^, while a class of salt taste-enhancing peptides can increase salt taste perception, but they by themselves do not have an intrinsic salt taste. Using papain to hydrolyze Harpadon nehereu (a species of lizardfish) proteins produced salty peptides that elicited salt taste intensity equivalent to that of 50 mM NaCl solution [69]. Using papain and Neutrase to hydrolyze Parapenaeopsis hardwickii (Spear shrimp) proteins produced salty peptides that had saltiness intensity equivalent to that of 10 mM and 55 mM NaCl, respectively [70]. Salty peptide fractions isolated from the protein hydrolysate of bovine bone when used in the range of 0.1–0.5 g/100 mL, produced saltiness intensity that was higher than that of NaCl at the same concentration [71]. Salty dipeptides (Ile-Gln, Pro-Lys, Ile-Glu, Thr-Phe, and Leu-Gln) have been identified in soy sauce [72]. In yeast extract, several salty peptides (Asp-Asp, Glu-Asp, Asp-Asp-Asp, Ser-Pro-Glu, and Phe-Ile) have been identified that also exhibit salt taste-enhancing effects [73]. In Chinese fermented soybean curds, four decapeptides were found to be the main taste-active compounds. A decapeptide (Glu-Asp-Glu-Gly-Glu-Gln-Pro-Arg-Pro-Phe) had the strongest salt taste-enhancing effect [74]. At present, the cellular mechanism and receptors that these peptides activate have not been elucidated.

## 5. Effect of TRPV1 Modulators on Neural and Behavioral Responses to Bitter, Sweet, and Umami Taste Stimuli

Bitter taste is detected by a subset of TRCs that express about 30 different G-protein coupled receptors, taste receptors type 2 (TAS2Rs). Sweet and umami tastes are detected by different subsets of TRCs that express G-protein-coupled taste receptors that are heterodimers of TAS1R2+TAS1R3 and TAS1R1+TAS1R3, respectively. Umami receptor is not only activated by glutamate, but this activation is strongly enhanced in the presence of 5′- ribonucleotides. The above receptors are coupled to the G-protein gustducin and require downstream signaling effectors PLCβ2, IP3R3, TRPM5/TRPM4, and voltage-gated ATP release heterooligomeric channel composed of CALHM1 and CALHM3 [63,75,76]. In addition, Na^+^-glucose symporter-1 is implicated in (TAS1R2+TAS1R3)-independent sugar sensing [77,78,79,80].

In geniculate ganglia, Cadherin (Cdh13)-expressing neurons receive information from bitter-sensing cells, Cdh4-expressing neurons receive information from umami-sensing cells, and Spondin1-expressing neurons receive information from sweet-tasting cells [6]. Bitter ganglion neurons connect to bitter taste receptor cells expressing Semaphorin 3A and sweet ganglion neurons connect to sweet taste receptor cells expressing Semaphorin 7A [75]. These data provide strong evidence that bitter, sweet, and umami taste are transduced via a labeled line. Thus, generation of different types taste cells from stem cells in the taste bud and their connections to specific ganglion cells are regulated by transcription factors [81].

### 5.1. TRPV1 and Bitter Taste

Genetic variation in TRPV1 and TAS2Rs influences ethanol sensations and may potentially influence how individuals initially respond to alcoholic beverages [82]. Activation of TAS2Rs augments capsaicin-evoked TRPV1 responses in rat pulmonary nociceptors involving phospholipase C and protein kinase C signaling pathway [83]. Trigeminal fibers associated with thermo-sensation and pain communicate with parabrachial taste neurons. This multisensory convergence is involved in interactions between gustatory and somatosensory hedonic representations in the brain [84].

Stimulating with quinine–HCl (10 mM) solutions containing RTX (1 or 10 μM), did not alter CT responses to quinine [22]. Similarly, quinine–HCl solutions containing I-RTX (1–100 nM) did not alter responses in CT, GL, and SL nerves [5].

Quinine is a representative bitter compound and its taste responses are entirely dependent upon the TAS2R-TRPM5 pathway. Similar to quinine, nicotine and ethanol are bitter and elicit an aversive response. In behavior studies, quinine was not aversive to TRPM5 KO mice, but nicotine was equally aversive in wildtype and TRPM5 KO mice. TRPM5 KO mice still showed residual CT responses to nicotine that were blocked by a nAChR antagonist, mecamylamine. In contrast to quinine, nicotine elicits taste responses through peripheral TRPM5-dependent pathways, common to other bitter tastants, and a nAChR-dependent and TRPM5-independent pathway [44]. The nAChRs are expressed in TRPM5-positive cells [85]. The taste of nicotine and mecamylamine mixtures was more similar to the taste of quinine than that of nicotine alone [44].

Nicotine activates Cap-sensitive trigeminal neurons [86,87]. To test if aversive responses to nicotine are dependent upon the trigeminal system, we injected Cap in neonate TRPM5 KO mice to produce systemic and life-long elimination of the majority of Cap-sensitive neurons [88]. Although responses to Cap solutions confirmed the treatment was effective, preference for 0.5- and 1.0-mM nicotine did not differ between untreated and Cap-treated KO animals. Thus, in addition to the TRPM5-dependent pathway, nAChRs serve as bitter taste receptors for nicotine [89,90], and the aversive response to nicotine is purely taste-mediated and is independent of the trigeminal system in the oral cavity.

In TRPM5 knockout (KO) mice, nAChR modulators (mecamylamine, dihydro-β-erythroidine, and CP-601932 (a partial agonist of the α3β4* nAChR)) inhibited CT responses to nicotine, ethanol, and acetylcholine. This suggests that nAChRs expressed in a subset of TRCs serve as common receptors for the detection of the TRPM5-independent bitter taste of nicotine, acetylcholine, and ethanol [91].

Sensory neurons from trigeminal or dorsal root ganglia as well as TRPV1-expressing HEK293 cells responded to ethanol in a concentration-dependent manner and are capsazepine-sensitive. Ethanol potentiated the response of TRPV1 to Cap, protons, and heat and lowered the threshold for heat activation of TRPV1 from approximately 42 °C to approximately 34 °C [92]. TRPV1 KO mice showed significantly higher preference for ethanol and consumed more ethanol in a two-bottle choice test as compared with wildtype littermates [93]. They also displayed reduced oral avoidance responses to ethanol regardless of concentration, insensitivity to Cap, and little to no difference in sweet or bitter taste responses relative to wildtype mice. These data indicate that TRPV1 plays a role in orosensory-mediated ethanol avoidance [94].

### 5.2. TRPV1 and Sweet Taste

Stimulating with sucrose (500 mM) containing RTX (1 or 10 μM) did not alter CT responses to sucrose [22]. Sucrose solutions containing I-RTX (1–100 nM) did not alter responses in CT, GL, and SL nerves [5]. Sweet taste receptor (T1R2+T1R3) responds to natural sugars, D-amino acids, sweet proteins, and artificial sweeteners. Saccharin, aspartame, acesulfame-K, and cyclamate activate TRPV1 in HEK 293 cell and dissociated primary sensory neurons, and sensitize TRPV1 channels to acid and heat in both systems. These results suggest that interaction of artificial sweeteners with TRPV1 may be involved in the off-taste of sweeteners [95].

### 5.3. TRPV1 and Umami Taste

Mice lacking Gα-gustducin, PLCβ_2_, IP_3_R_3_, and TRPM5 do not show large deficits in responses to umami taste stimuli [96,97,98,99]. These results suggest that glutamate is detected by multiple receptors and transduction pathways [100,101,102,103]. The candidate umami receptors include a variant of brain-expressed mGluR4, a heteromer (TASR1+TAS1R3), and a variant of the type 1 mGluR1 with a truncated NH_2_-terminal domain (truncated mGluR1).

TRPV1 KO mice elicited CT responses to MSG solutions containing Bz. Wildtype mice and rats elicited CT responses to MSG solutions containing Bz and SB-366791. In the presence of Bz+SB-366791, there is no contribution of Na^+^ to glutamate CT response [41]. In both wildtype and TRPV1 KO mice, IMP produced the same magnitude of increase in the CT response to glutamate. Thus, glutamate CT responses are TRPV1-independent.

## 6. Ligands with Dual Effects on Salty and Umami Tastes

Interestingly, some ligands that modulate salt responses also modulate responses to glutamate albeit at different concentration ranges. NGCC produced an effect on the Am-insensitive NaCl CT response between 1 and 5 μM. In contrast, between 10 μM and 40 μM, NGCC increased the CT response to MSG+Bz+SB-366791. Maximal enhancement was observed at 40 μM and increasing NGCC concentration to 60 or 100 μM did not further increase the response (Table 1). Adding 45 μM NGCC to chicken broth containing 60 mM Na^+^ enhanced the human umami taste intensity [55]. Although NGCC can directly activate hTRPV1, its effects on glutamate responses are TRPV1-independent. Increasing taste cell Ca^2+^ inhibited the NGCC-induced increase in CT response to glutamate but not the IMP-induced increase in glutamate response. This suggests that NGCC enhances umami taste by interacting with a Ca^2+^-dependent transduction pathway [55].

Xyl-MRP modulated Am-insensitive NaCl CT response between 0.1% and 0.5%. In contrast, Xyl-MRP at 2.5% or IMP significantly increased the CT response to MSG+SB366791+Bz (Table 1) [66]. Thus, the responses to glutamate and its enhancement in the presence of IMP and MRPs are indifferent to TRPV1 modulators.

Between 0.1 and 1.0%, FII_m_ produced a biphasic effect on NaCl+Bz CT responses. At 2.5%, FII_m_ enhanced the glutamate CT response equivalent to the enhancement with 1 mM IMP (Table 1). In human subjects, 0.3% FII_m_ produced enhancement of umami taste. These results suggest that FII_m_ modulates Am-insensitive salt taste and umami taste at different concentration ranges in rats and humans [64].

In HEK293T cells expressing hTRPV1, glutathione and γ-Glu-Val-Gly induced concentration-dependent responses similar to that of Cap. These responses were markedly attenuated by capsazepine, indicating that hTRPV1 may also be the target of kokumi taste stimuli (unpublished observations). Trigeminal ganglion cells co-express TRPV1 (sensitive to Cap) and TRPA1 (sensitive to allyl isothiocyanate). Intracellular Ca^2+^ imaging showed that pretreatment with γ-Glu-Val-Gly excited 7% of TG cells and increased the amplitude of their responses to allyl isothiocyanate, but not to Cap or menthol. The enhancing effect of γ-Glu-Val-Gly was prevented by a CaSR inhibitor. These results suggest that in cells expressing CaSR, TRPA1, and TRPV1, γ-Glu-Val-Gly preferentially activates TRPA1 [104].

## 7. Potential Binding Sites of Ligands to Taste Receptors Using *In Silico* Studies

Two kokumi peptides within yeast extract, IQGFK and EDFFVR, were shown to bind CaSR. IQGFK primarily interacted through electrostatic interactions, with key binding sites including Asp275, Asn102, Pro274, Trp70, Tyr218, and Ser147. EDFFVR mainly engaged via van der Waals energy and polar solvation free energy, with key binding sites being Asp275, Ile416, Pro274, Arg66, Ala298, and Tyr218 [105]. Gamma-glutamyl tripeptides (γ-Glu-Asn-Phe, γ-Glu-Leu-Val, γ-Glu-Leu-Tyr, γ-Glu-Gly-Leu, γ-Glu-Gly-Phe, γ-Glu-Gly-Tyr, γ-Glu-Val-Val, and γ-Glu-Gln-Tyr) induce kokumi taste by entering the Venus flytrap (VFT) of CaSRs and interacting with Ser147, Ala168, and Ser170. These peptides can enhance the umaminess of MSG as they can enter the binding pocket of the taste receptor type 1 subunit 3 (TAS1R3)–MSG complex [106]. Using a novel hypothetical receptor, taste type 1 receptor 3 (TAS1R3)–MSG complex constructed, kokumi-active γ-glutamyl peptides, four amino acid residues, Glu-301, Ala-302, Thr-305, and Ser-306 were critical in ligand–receptor interactions. These results demonstrated that kokumi-active γ-glutamyl peptides enhance the umami taste of MSG, and exhibit synergistic effects by activating TAS1R3 [107].

## 8. Summary

Acid responses are mediated via OTOP1 expressed in type III TRCs. TRPV1 is involved in responses to acids in the posterior oral cavity and larynx. The TRPV1 trigeminal system and the taste system work in concert to evoke aversive responses to acidic stimuli.

In HBO cells, while high salt increased δ-ENaC protein expression and decreased TRPV1 mRNA expression, Cap decreased δ-ENaC protein expression and increased TRPV1 mRNA expression. This suggests that, in HBO cells, TRPV1 regulates ENaC expression.

A variety of compounds of varying structures (Table 1) produce biphasic taste responses to NaCl in rodents and human subjects that are TRPV1-dependent.

The above compounds also produce biphasic response in CT response to KCl, NH_4_Cl, and CaCl_2_ that are also voltage-sensitive. This suggests the presence of a non-selective conductive pathway permeable to Na^+^, K^+^, NH_4_^+^, and Ca^2+^ in the anterior tongue.

OTOP1 also detects NH_4_Cl_._ Behavioral aversion to NH_4_Cl was completely abolished in mice that lacked both Type II cells and the OTOP1 channel in Type III cells. These results suggest that the aversive response to NH_4_Cl is not dependent upon the TRPV1 trigeminal system and resides entirely in Type II and Type III cells.

For the most part, neural and behavioral responses to sweet and umami taste stimuli are not affected by TRPV1 modulators. Artificial sweeteners also activate TRPV1. These results suggest that interaction of artificial sweeteners with TRPV1 may account for the off taste of sweeteners.

While most bitter tastants, including quinine, are transduced by the TAS2R-TRPM5-dependent transduction pathway, nicotine and ethanol are transduced by both a TAS2R-TRPM5-dependent and a TAS2R-TRPM5-independent pathway. The TAS2R-TRPM5-independent pathway is sensitive to nicotinic acetylcholine receptor antagonists. Both nicotine and ethanol activate TRPV1 trigeminal neurons. While aversive responses to ethanol are dependent upon the trigeminal system, the aversive responses to nicotine are not.

## 9. Future Directions

Future studies should focus on identifying non-selective cation conductance(s) in bitter, sour, and sweet cells that give rise to biphasic taste responses to NaCl, KCl, NH_4_Cl, and CaCl_2_ in the presence of TRPV1 modulators. Studies should be pursued to determine if TRPV1 modulators interact with OTOP1 or other acid detection pathways in circumvallate or foliate taste bud cells. It is important to further investigate TRPV1-dependent ENaC regulation in human salt-sensing taste cells by TRPV1 modulators, such as high salt and Cap. It is also imperative to examine the cellular mechanism by which some of the novel peptides described above elicit salt taste by themselves in the absence of Na^+^.

## Figures and Tables

**Table 1:** Effect of TRPV1 modulators on Am-insensitive NaCl CT response and Glutamate CT response.

TRPV1 Modulator	NaCl CT Response (Max Increase)	NaCl CT Response (Max Inhibition)	Glutamate CT Response Max Increase
Cetylpyridinium chloride	250 μM	2 mM	
CAP	40 μM	200 μM	
RTX	1 μM	10 μM	
NGCC	2.5 μM	50 μM	40 μM–100 μM
Nicotine	0.015 M	0.05 M	
Ethanol	40%	60%	
GalA-MRP	0.30%	1.0%	^§^ 2.5%
Kokumi peptides (FII_m_)	0.5%	1.0%	2.5%
Temperature	42 °C	55 °C	
pH_o_ + RTX	6	10	

Am-insensitive rat CT responses were recorded by stimulating the tongue with 100 mM NaCl+5 μM Bz in the presence of varying concentrations of TRPV1 modulators. Glutamate rat CT responses were recorded by stimulating the tongue with 100 mM MSG+5 μM Bz+1 μM SB-366791 in the presence of varying concentrations of TRPV1 modulators. ^§^ 2.5% Xyl-MRP.

## Abbreviations

Am	amiloride
Bz	benzamil
CALHM1	Ca^2+^ homeostasis modulator 1
CALHM3	Ca^2+^ homeostasis modulator 3
Cap	Capsaicin
CaSR	calcium-sensing receptor
CT	Chorda tympani taste nerve
CZP	Capsazepine
ENaC	epithelial Na^+^ channel
GalA	galacturonic acid, glucosamine
GL	glossopharyngeal nerve
GNAT3	guanine nucleotide-binding protein G(t) subunit alpha-3
HC-030031	2-(1,3-dimethyl-2,6-dioxopurin-7-yl)-N-(4-propan-2-ylphenyl)acetamide
HBO cells	cultured adult human fungiform taste cells
HEK	human embryonic kidney cells
IMP	inosine 5′ monophosphate
IP3R3	inositol triphosphate receptor type 3
I-RTX	iodo–resiniferatoxin
KO	knockout
mGluR4	metabotropic glutamate receptor 4
MRPs	Maillard-reacted peptides
MSG	monosodium glutamate
nAChR	nicotinic acetylcholine receptor
NGCC	N-geranyl cyclopropyl-carboxamide
NPS R568	N-(3-[2-chlorophenyl]propyl)-(R)-alpha-methyl-3-methoxybenzylamine
NPS-2143	2-chloro-6-[(2R)-2-hydroxy-3-[(2-methyl-1-naphthalen-2-ylpropan-2-yl)amino]propoxy]benzonitrile;hydrochloride
OTOP1	Otopetrin-1
pH_i_	intracellular pH
PIP2	phosphatidylinositol 4,5-bisphosphate
PLCβ2	phospholipase Cβ2
PKA	cAMP-dependent protein kinase A
PKD2L1	polycystin-2-like 1 channel
PKD1L3	Polycystin-1-like 3, transient receptor potential channel
RAAS	renin–angiotensin–aldosterone system
RTX	resiniferatoxin
SB-366791	*N*-(3-methoxyphenyl)-4-chlorocinnamide
SL	superior laryngeal nerve
TAS1R1, TAS1R2, TAS1R3	taste type 1 receptors
TAS2Rs	taste type 2 receptors
TRCs	taste receptor cells
TRPM5	Transient Receptor Potential Cation Channel Subfamily M Member 5
TRPM4	Transient Receptor Potential Cation Channel Subfamily M Member 4
TRPV1	transient receptor potential vanilloid type 1
TRPA1	transient receptor potential ankyrin 1
Xyl	xylose
